# Bryostatin-1: a promising compound for neurological disorders

**DOI:** 10.3389/fphar.2023.1187411

**Published:** 2023-06-07

**Authors:** Zhen Tian, Xin-Tong Lu, Xun Jiang, Jiao Tian

**Affiliations:** ^1^ College of Pharmaceutical Sciences, Southwest University, Chongqing, China; ^2^ Department of Pediatrics, Tangdu Hospital of Fourth Military Medical University, Xi’an, China; ^3^ Department of Infection, Children’s Hospital of Chongqing Medical University, National Clinical Research Center for Child Health and Disorders, Ministry of Education Key Laboratory of Child Development and Disorders, Chongqing Key Laboratory of Child Infection and Immunity, The First Batch of Key Disciplines on Public Health in Chongqing, Chongqing, China

**Keywords:** Alzheimer’s disease, Bryostatin-1, fragile X syndrome, multiple sclerosis, protein kinase C, stroke, traumatic brain injury

## Abstract

The central nervous system (CNS) is the most complex system in human body, and there is often a lack of effective treatment strategies for the disorders related with CNS. Natural compounds with multiple pharmacological activities may offer better options because they have broad cellular targets and potentially produce synergic and integrative effects. Bryostatin-1 is one of such promising compounds, a macrolide separated from marine invertebrates. Bryostatin-1 has been shown to produce various biological activities through binding with protein kinase C (PKC). In this review, we mainly summarize the pharmacological effects of bryostatin-1 in the treatment of multiple neurological diseases in preclinical studies and clinical trials. Bryostatin-1 is shown to have great therapeutic potential for Alzheimer’s disease, multiple sclerosis, fragile X syndrome, stroke, traumatic brain injury, and depression. It exhibits significant rescuing effects on the deficits of spatial learning, cognitive function, memory and other neurological functions caused by diseases, producing good neuroprotective effects. The promising neuropharmacological activities of bryostatin-1 suggest that it is a potential candidate for the treatment of related neurological disorders although there are still some issues needed to be addressed before its application in clinic.

## 1 Introduction

**FIGURE 1:**
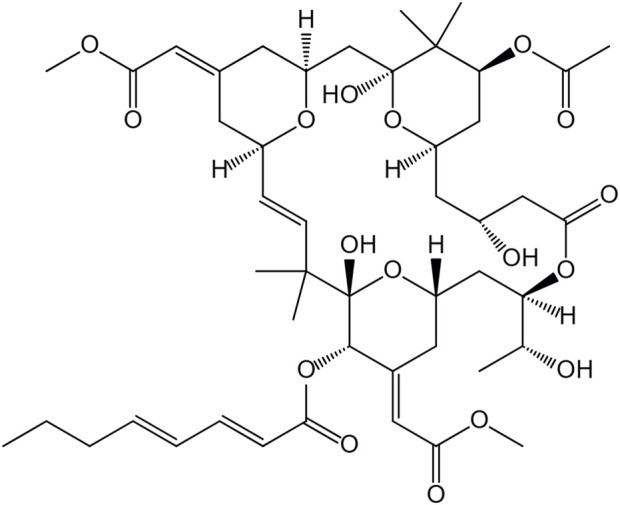
The chemical structure of bryostatin-1.

The central nervous system (CNS) is the most complex system of human body with a variety of functions still being unclear. “CNS diseases” is often refereed as an umbrella term for a multiple of disorders affecting either the spinal cord or brain or both such as neurodegenerative diseases, neurovascular disorders (e.g., hemorrhages, stroke), infections (e.g., meningitis, encephalitis), neuropsychiatric disorders (e.g., depression, schizophrenia), structural disorders (e.g., brain or spinal cord injuries), or conditions such as migraines, epilepsy, *etc.* (GBDN, 2019). Generally speaking, the pathophysiology of these CNS diseases is often very complex and largely unknown. Taking neurodegenerative diseases as an example, they are mainly caused by the selective dysfunction and progressive degeneration of neurons, glial cells, synapses, and interconnected networks. Currently, the effective treatment strategies for CNS diseases are quite limited due to our inadequate understanding of the underlying mechanisms of these diseases. The current medications only provide marginal benefits rather than halting the disease progression. Natural compounds with multiple pharmacological activities may offer better options for the remedy of neurological disorders because they have broad cellular targets and potentially produce synergic and integrative effects (Leon et al., 2013). Marine organisms are important and exciting sources of unique bioactive natural products. A series of clinically used drug candidates, such as vidarabine, omega-3-acid ethyl esters, eribulin and monomethyl auristatin, are derived from marine organisms directly or indirectly (Malve, 2016; Newman and Cragg, 2016). The bryostatins are a group of highly oxygenated marine macrolides with a unique polyacetate backbone separated from marine invertebrate animals. In 1970s, Petit *et al* found that extracts of the marine organism Bryozoan *Bugula neritina* had anticancer activity, which further led to isolating bryostatin-1 as a key metabolite in 1982 (Figure 1). To date, a total of more than twenty natural bryostatins (bryostatin 1–21) have been found. Bryostatins have attracted much attention because of their extensive bioactivities in recent years. Among the family of bryostatins, bryostatin-1 is the flagship member and also the most frequently investigated substance. Bryostatin-1 has remarkably diverse biological activities and is demonstrated to be promising in a variety of preclinical and clinical studies for cancer (Kortmansky and Schwartz, 2003), Alzheimer’s disease (AD) (Etcheberrigaray et al., 2004; Farlow et al., 2019), HIV (Wender et al., 2017), diabetes (Way et al., 2001), stroke (Sun et al., 2008), multiple sclerosis (Kornberg et al., 2018) and some other diseases (Zuo et al., 2019).

Bryostatin-1 has been shown to produce various biological activities through binding with the protein kinase C (PKC). The rings of bryostatin-1 form a cap-like structure upon binding with the N-terminal C1 domain of PKC. Despite its hydrophilicity, bryostatin-1 exhibits a high affinity for PKC with potency similar to phorbol ester (a typical hydrophobic ligand of PKC) (Mochly-Rosen et al., 2012). PKC is a family of serine/threonine kinases that can phosphorylate its substrates through transferring the γ-phosphate group of ATP to the hydroxyl groups of serine and threonine amino acid residues. PKC plays important roles in a variety of cellular processes such as proliferation, differentiation, migration and cell survival through regulating multiple signal transduction events. The dysfunction of PKC is closely associated with the pathophysiology of many diseases like cancer (Griner and Kazanietz, 2007), cardiovascular diseases (Murphy and Frishman, 2005) and neurological disorders (Etcheberrigaray et al., 2004). PKC is categorized into three subfamilies based on their structural and activation characteristics. The classical PKC (cPKC) subfamily contains four isoforms: PKCα, PKCβ1, PKCβ2 and PKCγ. The novel PKC (nPKC) subfamily is composed by PKCδ, PKCε, PKCη, PKCθ, while the atypical PKC (aPKC) subfamily consists of PKCτ/λ and PKCζ (Steinberg, 2008; Newton, 2018).

Although bryostatin-1 can bind to multiple PKC subtypes, the binding affinities towards different subtypes are diverse. The binding affinities of bryostatin-1 with PKCα, PKCβ2, δ, and ε isoforms are 1.35, 0.42, 0.26, and 0.24 nM, respectively (Szallasi et al., 1994; Raghuvanshi and Bharate, 2020). In cultured neuronal cells, bryostatin-1 was found to induce potent PKCα, δ, and ε activation at 10^–8^, 10^–9^, and 10^–10^ M. Time course experiments showed that 10^–10^ M bryostatin-1 triggered significant PKCε and PKCδ activation by 30 min and 1 h, respectively (Yi et al., 2012). Bryostatin-1 appears to have a greater specificity toward PKCε and PKCδ isoforms, especially the former. The preference of bryostatin-1 for PKCε over other isoforms was also observed *in vivo* study (Nelson et al., 2014). After binding, bryostatin-1 induces the self-phosphorylation and activation of PKCs rapidly by promoting its membrane translocation, whereas a prolonged interaction of bryostatin-1 with PKCs can also lead to downregulation and inhibition of PKCs (Mochly-Rosen et al., 2012). There are characteristically three phases in the time course of PKC activity, with activation for less than 40 min, following by a downregulation phase for several hours and then the phase of *de novo* synthesis (>2 days) (Sun et al., 2008; Farlow et al., 2019). The activation of PKC is attributed to the translocation of cytoplasmic enzyme to cellular membrane in response to second messengers. Subsequent ubiquitination and proteasome degradation of the enzyme accounts for the downregulation phase, which is followed by recycling of degraded products into *de novo* synthesis of the original enzyme (Sun et al., 2008; Farlow et al., 2019).

PKC is abundant in the central nervous system and plays critical roles in maintaining normal brain function. Among the PKC subtypes, PKCα, PKCγ, PKCε and PKCζ are sometimes referred to as “memory kinases” because of their vital function in the cellular mechanisms underlying learning and memory (Sun and Alkon, 2014). Bryostatin-1 has exhibited promising neurological activity in several rodent models of brain disorders. Bryostatin-1 was shown to be effective in rescuing spatial learning and memory deficits of animals with AD (Hongpaisan et al., 2011), fragile X syndrome (Sun et al., 2014) and ischemic stroke (Sun et al., 2009). Mechanism study revealed that bryostatin-1increased BDNF level in the hippocampus (Sun et al., 2014) and facilitates hippocampal long-term potentiation (Kim et al., 2012). In addition, it increased hippocampal dendritic spine density and mushroom spine number (Hongpaisan and Alkon, 2007; Hongpaisan et al., 2013), and prevented the spine and synapse loss (Hongpaisan et al., 2011). The promising neuroprotective effects of bryostatin-1 make it possible to be an alternative medicament for treating the relevant diseases. However, the common limitations of using natural compounds as drugs, such as inconsistent potency, low purity, and poor stability, can still not be neglected in clinical transformation of bryostatin-1. On the other hand, some special drawbacks including low natural abundance, difficulty in total synthesis, and potential toxicity, may also hinder the widely use of bryostatin-1. Based on all of the above, an urgent need to thoroughly understand the true face of bryostatin-1 has emerged. In this review, we mainly summarized the pharmacological activities of bryostatin-1 in treating neurological disorders, and discussed its pharmacokinetic characteristics and safety profile as well. The synthesis of these scientific evidences about neuroprotective properties of bryostatin-1 could facilitate future research to further explore the feasibility of bryostatin-1 in clinical therapy.

## 2 Neuroprotective effects of bryostatin-1

### 2.1 Bryostatin-1 and Alzheimer’s disease

#### 2.1.1 The therapeutic potential of bryostatin-1 in AD

Alzheimer’s disease (AD) is a common neurodegenerative disorder with few effective treatment options. Today, nearly 55 million people suffer from AD globally, and this number is estimated to be approximately 152 million by 2050 (Janoutova et al., 2021). In developed countries, about one-tenth of elderly people (above 65 years old) are affected by AD in its early stages, and over one-third of very elderly people (above 85 years old) may have advanced symptoms (Qiu et al., 2009). AD consumes a large amount of medical resources and brings a huge burden to the society. The costs related with AD is estimated to be about $305 billion according to a report in 2020, and it is expected to exceed $1 trillion in 2050 (Wong, 2020). Currently, cholinesterase inhibitors (such as donepezil, tacrine and galantamine) and N-methyl-D-aspartate receptor antagonists (i.e., memantine) are the main drugs used to treat AD (Tayeb et al., 2012). However, both drug classes offer only marginal benefits, therefore more effective therapies are still needed. The deposition of extracellular “plaques” containing β-amyloid (Aβ) and the aggregation of neurofibrillary tangles consisting of hyper-phosphorylated tau protein are the pathological hallmarks of AD, which are thought to provoke progressive neurodegeneration as well as behavioral and cognitive impairment in AD (Zlokovic, 2005). The proteolytic processing of amyloid precursor protein (APP) by β- and γ-secretases leads to the aggregation of Aβ, while α-secretase cleavages APP into soluble APPα (sAPPα), which is not only non-toxic but also inhibits the formation of amyloid plaques. The imbalance between Aβ production and clearance contributes to the aggregation of neurotoxic Aβ. Thus, it is a potential strategy to attenuate synapse loss and cognitive dysfunction in AD through preventing oligomeric Aβ accumulation.

There is a negative feedback loop between Aβ formation and PKC activation. Aβ is shown to decrease brain PKC levels and activity (Lee et al., 2004; Liron et al., 2007; Hongpaisan et al., 2011), while PKC hypofunction conversely leads to the defects of APP processing and significantly contributes to AD pathophysiology and progression (Pascale et al., 1998). The modulation of PKC activity, particularly the PKCδ and PKCε isoforms, is one potential effective way to trigger α-secretase processing of APP, producing non-toxic sAPPα instead of toxic Aβ, preventing senile plaque formation and AD-associated cognitive deficits (Yi et al., 2012). For example, PKCε activation was also shown to promote Aβ degradation by increasing endothelin converting enzyme (ECE) activity besides its inhibition in Aβ accumulation through enhancing α-secretase dependent anti-amyloidogenic pathway (Choi et al., 2006; Nelson et al., 2009). As a potent PKC activator, bryostatin-1 can effectively activate PKCδ and ε isozymes at sub-nanomolar concentrations (Tanabe et al., 2007). The potent and sustained activation of PKC by bryostatin-1 leads to the rapid and persistent activation of α-secretase APP processing, then preventing the aggregation of Aβ (Yi et al., 2012). Recently, a paper showed that nanoparticle-encapsulated bryostatin-1 displayed greater potency in the activation of PKC and α-secretase than the unmodified form (Schrott et al., 2020), indicating that the form of bryostatin-1 may be an important factor affecting the efficacy. The effects of bryostatin-1 on Aβ generation and clearance suggested that it may represent an important, novel, and specific treatment for AD.

Actually, the therapeutic potential of bryostatin-1 in AD has been investigated in a variety of preclinical and clinical studies. Long-term and short-course administration of bryostatin-1 both showed protective effects in the mouse models of AD. The administration of bryostatin-1 intraperitoneally (i.p.) at a dose of 30 μg/kg twice a week for 12 weeks dramatically prevented synaptic loss, inhibited Aβ accumulation and improved the cognitive function of APP/PS1 mice (Hongpaisan et al., 2011). Administration of bryostatin-1 orally (5 μg each time) for three alternative days during a pretreatment week and then daily during the second “testing” week significantly reduced the latency and distance of APP/PS1 mice to find the escape platform in Morris water maze test. It indicated that acute oral administration of bryostatin-1 can also remarkably improve the learning ability and cognitive function of AD transgenic mice in a relatively shorter (1–2 weeks) treatment period (Schrott et al., 2015). Bryostatin-1 also markedly enhanced the spatial learning and memory ability of 5XFAD mice, another AD transgenic mouse model, and its effect can be mimicked by PKC ε specific activator DCP-LA (Hongpaisan et al., 2011).

Owing to its promising effects in animal models, bryostatin-1 entered clinical trials for treating AD. In a single-dose (25 μg/m^2^) randomized double-blind Phase IIa clinical trial, six of nine patients diagnosed as AD received bryostatin-1 and the other three received placebo. It was found that bryostatin increased the Mini-Mental State Examination (MMSE) score by 1.83 ± 0.70 unit *versus* −1.00 ± 1.53 unit for placebo at 3 h of injection (Nelson et al., 2017). In another Randomized, Double-Blind, Placebo-Controlled, Phase II study, advanced AD patients were randomized equivalently into 20 μg and 40 μg bryostatin-1, and placebo arms. Bryostatin-1 or placebo was given (i.v. Over 45 ± 5 min every time, with a total of 7 doses) to patients over a period of 12 weeks. Although the primary improvement of Severe Impairment Battery (SIB) scores at week 13 was not significant in the Full Analysis Set (FAS), the SIB comparison favored 20 μg bryostatin-1 compared to placebo patients in the Completer Analysis Set (CAS). Moreover, the SIB improvement by bryostatin-1 could still be observed at week 15, i.e., several weeks after the termination of the dosing protocol (Farlow et al., 2019). In two double-blind placebo-controlled Phase II trials, bryostatin-1 showed potential efficacy in pre-specified cohorts with advanced AD patients, in the absence of memantine. This efficacy involved improvement of 4.0 points or more in the SIB, without any side effects compared to the placebo patients (Thompson et al., 2022).

#### 2.1.2 Bryostatin-1 inhibits Aβ accumulation

Among the pathologic features of Alzheimer’s disease (AD), synaptic loss is mostly correlated with dementia degree of AD patients. Aβ is the main driving force to induce synaptic loss. Reduced PKCα/ε activity and/or expression were found in brains of AD patients (Matsushima et al., 1996; Favit et al., 1998). And this reduction of PKC isozymes in AD brains was closely related with Aβ-induced synaptic loss. In contrast, activation of synaptogenic PKCε was found to prevent amyloid plaque formation, early synaptic loss and cognitive deficits in AD transgenic mice (Hongpaisan et al., 2011).

Bryostatin-1 was found to block the elevation of soluble Aβ_42_ protein in the extranuclear cell body compartment and dendrites of the hippocampal CA1 pyramidal neurons of Tg2576 mice (Hongpaisan et al., 2011). It also suppressed PKCε reduction within the presynaptic compartments of CA3 neurons, prevented the loss of PKC ε-containing axonal boutons and the reduction of PKCα-positive area in CA1 pyramidal neurons through reducing Aβ protein level. The inhibition of bryostatin-1 in Aβ aggregation and positive modulation of PKC activity remarkably improved the learning and memory performance of AD mice (Hongpaisan et al., 2011). ADAM10 is a member of the ADAM (*A Disintegrin And Metalloproteinase*) family, and was found to act as constitutive α-secretases in the non-amyloidogenic processing of the amyloid-β protein precursor (AβPP) and generate neuroprotective sAβPPα peptide instead of toxic oligomeric Aβ proteins (Kuhn et al., 2010). The expression of ADAM10 is found to be decreased by Aβ_40_ treatment in the cultures of SH-SY5Y cells. Bryostatin-1 was shown to promote sAβPPα generation via restoring ADAM10 expression and exert neuroprotective effects *in vitro* (Marchesi et al., 2016). Neprilysin (NEP) is a major Aβ peptide-degrading enzyme and responsible for the clearance of Aβ in the brain (Shirotani et al., 2001). The expression and activity of NEP was reduced in the AD brain (Reilly, 2001), then up-regulating NEP may inhibit Aβ accumulation and prevent synapse loss and cognitive impairments. PKCε activation enhances HuD expression and facilitates its post-transcriptional regulation of target genes (Pascale et al., 2005). Bryostatin was shown to increase NEP association with HuD and elevate NEP mRNA stability, as well as upregulate NEP expression and activity through activation of PKCε, which then promotes the degradation of oligomeric Aβ protein (Lim and Alkon, 2014).

#### 2.1.3 Bryostatin-1 inhibits oxidative stress

Oxidative stress is another important factor provoking the neurodegeneration of AD. Reduced expression of manganese superoxide dismutase (MnSOD) and increased of Aβ level was observed in hippocampal neurons from autopsy-confirmed AD patients (Sen et al., 2018). In cultured human primary hippocampal neurons, PKCε knockdown led to the accumulation of Aβ and the production of reactive oxygen species (ROS), which then further inhibited the activity of PKCε and shaped a vicious circle (Sen et al., 2018). The reduction of PKCε and subsequent oxidative stress can be prevented by bryostatin-1 in both cultured neurons and Tg2576 AD transgenic mice (Sen et al., 2018). Cerebral vascular endothelial dysfunction and capillary loss are the main reasons for brain microcirculation disturbance and they are also primary phenotypes of normal aging and AD (Brown and Thore, 2011; Ungvari et al., 2018). The results of autopsy showed that there was a significant decrease in the expression of PKCε, MnSOD and vascular endothelial growth factor (VEGF) as well as microvascular density in the hippocampus of AD patients (Millien et al., 2022). Bryostatin also increased the expression of PKCε, MnSOD, VEGF and attenuated oxidative stress in cultured human brain microvascular endothelial cells (Millien et al., 2022). In aged rats (>24 months old) and Tg2576 AD transgenic mice (5 months old), bryostatin both blocked the reduction of vascular PKCε, MnSOD, and VEGF and attenuated microvascular loss and memory defects (Millien et al., 2022).

#### 2.1.4 Bryostatin-1 promotes synaptogenesis

Changes in dendritic spine and synapse density are believed to underlie the effects of procognitive drugs. PKCε is highly expressed in presynaptic nerve fibers, indicating an important role in neurite outgrowth, synapse formation and neurotransmission (Shirai et al., 2008). Both PKC ε and PKC α are crucial for synaptic proteins synthesis and their activation can facilitate synaptogenesis and suppress synaptic loss under pathological conditions (Hongpaisan and Alkon, 2007; Sun et al., 2008).

BDNF plays a key role in synaptogenesis and it can be activated by PKC. The suppression of BDNF resulted from hypofunction of PKCε and PKCα devotes to the decrease of presynaptic axonal boutons and postsynaptic dendritic spines, as well as synaptic loss at early stage of AD. Bryostatin-1 was shown to suppress the reduction of BDNF in the hippocampus of Tg2576 mice (Hongpaisan et al., 2011). Bryostatin-1 was shown to promote the accumulation of Hu proteins in the dendritic shafts, which then enhanced the synthesis of synaptic proteins (e.g., synaptophysin, spinophilin) and increased presynaptic vesicles, “perforated” postsynaptic densities as well as double-synapse presynaptic boutons in the rat hippocampus (Hongpaisan and Alkon, 2007). Bryostatin-1 increased synapse density in primary cultured cortical neurons with inverted U-shaped concentration and time responses (Ly et al., 2020). This biphasic responses caused by bryostatin-1 may be attributed to the fact that bryostatin-1 could induce ubiquitination-depended PKC downregulation at higher concentration or prolonged incubation (Lee et al., 1997). Its effects could be blocked by PKC inhibitor or inactive structural analogues of bryostatin-1, indicating that the promoting effect of bryostatin-1 on synaptogenesis in cortical cultures was indeed mediated by PKC (Ly et al., 2020). The promoting effects of bryostatin-1on mushroom spine synapses and total number of synapses were also demonstrated in Tg2576 mice via restoring PKCα and PKCε function, which contributes to the improvement of memory deficits in AD transgenic mice (Hongpaisan et al., 2011).

Dendritic spines are key postsynaptic structures where the majority of PSD-95 is localized. However, bryostatin-1 significantly reduced dendritic spine density in cortical neurons, with a greater reduction in the density of filopodia spine than mushroom spine. Its effects on spine density can be blocked by PKC-inactive analogues or its inhibitor (Ly et al., 2020). Dendritic spine density largely determines dendritic arbor complexity. However, bryostatin-1 had no effect on the dendritic arbor complexity of cultured cortical neurons. That is to say, bryostatin-1 does not affect dendritic branching, while increases cortical synaptogenesis with a concomitant decrease in cortical spine density in a PKC-dependent pathway (Ly et al., 2020). It seems that bryostatin-1 enhances synaptic density while reduces immature dendritic spine density and thus improves cortical communication via enhancing the signal-to-noise ratio. Actually, the effect of bryostatin-1 on neurotransmission has been tested by electrophysiological recording. Bryostatin-1 markedly increased the amplitude and frequency of spontaneous inhibitory postsynaptic currents (sIPSCs), the firing rate of GABAergic interneurons as well as the paired-pulse ratio of GABAergic synapses in the hippocampus of Brown Norway rats. The effects of bryostatin-1 on GABAergic transmission can be blocked by PKC antagonist (Xu et al., 2014), suggesting that bryostatin-1 enhanced hippocampal GABAergic neurotransmission via PKC activation. These synaptic remodeling and synaptogenesis provide structural and functional storage sites for long-term associative memory, and may explain why bryostatin-1 is effective in improving spatial learning and memory (Hongpaisan and Alkon, 2007).

### 2.2 Bryostatin-1 and multiple sclerosis

Multiple sclerosis (MS) is a neurodegenerative disease and also the second leading cause of disability in young adults, which is characterized by persistent neuroinflammation and subsequent demyelination (Wingerchuk and Carter, 2014). It is considered to be a multifactorial disease and the exact pathogenesis is still obscure. In most patients (over 80%), the initial stage of this disease is characterized by neurologic disability resulted from focal inflammation, which can achieve complete remission with leaving no or only minor sequelae after intervention (also referred as relapsing-remitting MS, or RRMS). Subsequently, many patients gradually develop into secondary progressive MS, which cannot be completely alleviated after the recurrence, with the neurologic disability gradually worsening (Dutta and Trapp, 2014). The inflammatory process involving the activation of multiple immune cells is closely related with the initiation and progression of MS. The inflammatory plaques of RRMS are mainly composed of Th cells, which infiltrates from the periphery to CNS and are restimulated by local myeloid cells such as microglia and macrophages. In progressive forms of MS, there is continuous innate myeloid cell activation at the edges of gradually expanding plaques as well as the whole white and gray matter though the infiltration of lymphocytic cells no longer exists (Lassmann et al., 2012).

Treatment with bryostatin-1 (i.p.) starting from the day of immunization prevented the onset of neurologic deficits induced by MOG_35-55_ in the mouse model of experimental autoimmune encephalomyelitis (EAE). It also significantly reduced the number of total CD4^+^, Th1 and Th17 lymphocytes in the lymph nodes of mice with MOG_35-55_ immunization. Bryostatin-1 not only inhibited the peripheral immune response but also suppressed the infiltration of CD4^+^ lymphocytes into the brain and spinal cord (Kornberg et al., 2018). More strikingly, bryostatin-1 could still attenuate neurologic deficits after EAE onset, even when treatment began at day 28 postimmunization (more than 10 days after peak disease) (Kornberg et al., 2018). Intravenous administration (i.v.) of bryostatin-1 (carried by engineered extracellular vesicles) every 3 days from day 14 postimmunization (disease peak) for 15 days remarkably reduced the percentage of demyelination area and decreased the total number of mononuclear cells, the proportions and absolute numbers of CD4^+^ cells in the spinal cord and brain of EAE mice (Wu et al., 2022a). Astrogliosis is a main feature of demyelinating lesions, which can prevent remyelination and aggravate MS disease through the induction of glial scarring (Reynolds et al., 2011; Li et al., 2016). Bryostatin-1 treatment significantly alleviated astrogliosis in the spinal cord of EAE mice (Wu et al., 2022a). Microglia activation may also aggravate the demyelinating dysfunction of multiple sclerosis. The results of microglia staining in the spinal cord showed that the percentage of pro-inflammatory M1 phenotype decreased while that of anti-inflammatory M2 phenotype increased following bryostatin-1 treatment. It indicated that bryostatin-1 could promote the transformation of microglia from proinflammatory phenotype to neuroprotective phenotype (Wu et al., 2022a). Bryostatin-1 was also shown to inhibit the expression of multiple inflammatory cytokines that played crucial roles in the induction of Tregs and Th17 cells in the spinal cord (Koehler et al., 2008). These results suggested that bryostatin-1 was able to suppress CNS inflammation effectively.

The neuroprotective effects of bryostatin-1 were also demonstrated in other demyelinating animal model. In the mouse model induced by cuprizone (mixed in standard chow and fed for 4 weeks), administration of bryostatin-1 (i.v., encapsulated by exosomes) every 3 days starting at fifth week for 2 weeks effectively prevented the myelin injury and increased the area of myelination (Wu et al., 2022b). Bryostatin-1 treatment also markedly attenuated the destruction of myelin sheaths and reduced the gap between myelin layers, as well as increased the proportion of oligodendrocyte remyelinated axons (Wu et al., 2022b). Consistent with the function of accelerating myelin regeneration, bryostatin-1 also remarkably increased the myelin thickness of remyelinated axons (Wu et al., 2022b). Similarly, bryostatin-1 also inhibited the activation of astrocytes in the corpus callosum of cuprizone-treated mice (Wu et al., 2022b). Additionally, bryostatin-1 treatment downregulated iNOS expression but increased the Arg1 expression in microglia, suggesting that it can promote the switching of microglia from pro-inflammatory phenotype to neuroprotective phenotype (Wu et al., 2022b). Apart from the neuroinflammation, oxidative stress, enhanced matrix metalloproteinases (MMPs) activity and blood–brain barrier (BBB) damage also significantly contributed to the pathogenesis of MS (Safaeinejad et al., 2018). Bryostatin-1 was also shown to possess the activity of antioxidation and MMP inhibition, which was helpful for the recovery of MS (Safaeinejad et al., 2018). Taken together, the above data suggested that bryostatin-1 has a great potential to be developed as a therapeutic agent for MS, especially for progressive forms of MS.

### 2.3 Bryostatin-1 and fragile X syndrome

Fragile X syndrome (FXS) is a common inherited cause of intellectual disability as well as the primary monogenetic cause of autism spectrum disorder (ASD) (Belmonte and Bourgeron, 2006). FXS is resulted from the expansion of a CGG repeat in the promoter region of the fragile X messenger ribonucleoprotein 1 (*Fmr1*) gene, which then caused the loss of fragile X messenger ribonucleoprotein (FMRP) (O'Donnell and Warren, 2002). FMRP is shown to affect the translation of multiple mRNAs that are critically involved in the regulation of synaptic plasticity and synaptic maturation (Banerjee et al., 2018). The lack of FMRP contributed significantly to the disordered brain architecture and dysregulated synaptic function (Bagni and Zukin, 2019), which may be the pathological mechanisms underlying FXS and its concomitant disorders such as depression, cognitive deficits, attention disturbance, hyperactivity (Hagerman et al., 2018). So far, there is still lacking effective treatment strategy for FXS.

Chronic administration of bryostatin-1 (i.v.) through tail vein for 13 weeks (20 μg/m^2^, two doses every week), a timescale resembles years of treatment in humans, inhibited hyperactivity of *Fmr1* KO mice, a mouse model recapitulating behavioral symptoms of humans with FXS (Cogram et al., 2020). Bryostatin-1 also normalized the nesting behavior and marble burying behavior in the *Fmr1* KO mice, which depended on the integrity of the hippocampal circuit (Cogram et al., 2020). More importantly, bryostatin-1 for 13 weeks attenuated the impairments of contextual fear conditioning memory (Cogram et al., 2020) and the deficits of spatial learning and memory of the FXS model mice (Sun et al., 2014). However, treating the *Fmr1* KO mice with bryostatin-1 for 5 weeks, a timescale comparable to several months of human treatment, had very limited or no therapeutic effects on the behavioral and cognitive deficits (Cogram et al., 2020). It indicated that the therapeutical effects of bryostatin-1 on FXS need an enough longer period to appear.

It has been shown that chronic bryostatin-1 administration for 13 weeks (20 μg/m^2^, two doses/week) markedly elevated the expression of BDNF and PSD95 in the hippocampus of adult *Fmr1* KO mice (Sun et al., 2014). Morphological results showed that bryostatin-1 increased the density of mature and overall dendritic spines but decreased the density of immature spines, indicating that bryostatin-1 promoted the maturation of dendritic spines (Sun et al., 2014). Bryostatin-1 also suppressed the reduction of presynaptic vesicles concentration and synapses density in the hippocampal CA1 stratum radiatum of *Fmr1* KO mice (Sun et al., 2014). The results suggested that long-term treatment with bryostatin-1 could indeed alleviate the synaptic and cognitive dysfunction of adult FXS mice. A relative shorter term administration of Bryostatin-1 (20 μg/m^2^, tail vein i.v., two doses/week for 6 weeks) on young *Fmr1* KO mice (starting at an age of close to 4 weeks) was also investigated (Sun et al., 2016). The young *Fmr1* KO mice also showed spatial learning and memory impairments similar as the adult mice, which may be attributed to the reduced BDNF expression and PSD-95 accumulation, decreased mushroom-shape dendritic spine density as well as impaired synaptic maturation in the apical dendrites of hippocampal CA1 neurons (Sun et al., 2016). Bryostatin-1 treatment can also rescue the synaptic dysregulation and cognitive dysfunction of young *Fmr1* KO mice. These data suggest that the younger patients may still benefit from the shorter term bryostatin-1 treatment despite its limited effects on adult patients.

### 2.4 Bryostatin-1 and stroke

Stroke is the second-leading cause of death and the third-leading cause of disability globally (Stocchetti et al., 2015). Ischemic stroke is the most common type of stroke, which is resulted from cerebrovascular occlusion and characterized by a sudden reduced or cut-off of blood flow to the brain regions. At present, recombinant tissue plasminogen activator (rt-PA) remains the only drug approved by Food and Drug Administration for treating acute ischemic stroke. However, only a very small proportion of ischemic patients benefit from rt-PA due to its short time-window and increased risk of secondary cerebral hemorrhage (Katzan et al., 2004). The strategies that can reduce the risk of hemorrhagic transformation induced by rt-PA and extend its time-window may be beneficial for treating stroke.

In the aged female rats suffering from middle cerebral artery occlusion (MCAO), administration of bryostatin-1 (i.p.) at 2 h and r-tPA at 6 h after the surgery significantly improved the survival rate and inhibited cerebral swelling, indicating that bryostatin-1 may extend the time-window of r-tPA (Tan et al., 2015). Coadministration of bryostatin-1 and rt-PA also led to a further decrease in the relative hemoglobin concentrations in cortex, striatum and total cerebral hemisphere at 24 h post-MCAO, implying that bryostatin-1 suppressed the hemorrhagic transformation following ischemia (Tan et al., 2015). The activation of matrix metalloproteinase-9 (MMP-9) leads to the degradation of basal lamina matrix proteins and the proteins of the extracellular matrix, which then causes BBB disruption and swelling formation (Rosenberg et al., 1998). The BBB disruption and MMP activation-induced basement membrane remodeling may enhance the risk of hemorrhagic transformation following ischemia (Wang and Lo, 2003), which contributes to post-stroke morbidity and mortality. The coadministration of bryostatin-1 and rt-PA led to a greater reduction of MMP-9 activity compared to rt-PA treatment alone (Tan et al., 2015). The upregulation of PKCε activity by bryostatin also potently inhibited the damage of tight junctions within the BBB and then reduced the risk of hemorrhagic transformation following reperfusion by rt-PA (Tan et al., 2015).

The protective effects of bryostatin-1 against stroke were also observed by other groups. The results of Tan *et al* suggested that administration of bryostatin-1 (i.p.) at 6 h post-MCAO, then at 3, 6, 9, 12, 15, and 18 days after MCAO improved the survival rates of model rats through 21 days experimental schedule. There was also a significant neuro-functional improvement in the bryostatin-treated rats at 21 days post-MCAO. Bryostatin-1 reduced the latency speed and distance traveled of ischemic rats to the platform in Morris water maze test (Tan et al., 2013). Morphological results showed that bryostatin-1 treatment reduced the lesion volume of cortex, striatum, and total hemisphere at 21 days post-MCAO as well as inhibited the cortical hemispheric swelling or atrophy (Tan et al., 2013). In a global cerebral ischemic rat model, bryostatin-1 was given (15 μg/m^2^, i.v., two doses/week for 5 weeks) with the first dose administered 24 h after the end of the cerebral ischemia. It displayed persistent improvement on the spatial learning and memory abilities at least for another 4 months after the last administration (Sun et al., 2009). It suggested that postischemic administration of bryostatin-1 could produce a lasting functional recovery of neural circuits. Another group found that co-administration of PKC isozyme inhibitor Ro-31-8220 significantly attenuated the restoring effects of bryostatin-1 on the learning and memory, indicating that the function of bryostatin-1 depends on the PKC activation (Sun et al., 2008).

The neuronal apoptosis and gradual loss of synapses in the dorsal hippocampus contributed to the impairment of spatial learning and memory caused by ischemia (Sun et al., 2008). Administration of bryostatin-1 for 5 weeks significantly enhanced BDNF activity and inhibited the neuronal apoptosis in the dorsal hippocampal CA1 of ischemic rats (Sun et al., 2008; Sun et al., 2009). Moreover, bryostatin-1 also suppressed the cell apoptosis in the peri-infarct region and attenuated astrocyte activation following MCAO (Tan et al., 2013). As we know, long-term memory is stored in the brain via the formation of mushroom-shaped dendritic spines-containing perforated postsynaptic density (PSDs) (Nagerl et al., 2007). These mushroom spines form multiple synapses with preexisting axonal boutons, which have already shaped synapse with other dendritic spines (Geinisman et al., 2001; Hongpaisan and Alkon, 2007). Bryostatin-1 rescued ischemia-induced decrease of dendritic spines, presynaptic vesicles as well as mushroom and stubby spine formation in the dorsal hippocampal CA1 area of ischemic rats (Sun et al., 2008). In addition, the loss of dendritic spine synapses caused by cerebral ischemia was also attenuated by chronic bryostatin-1 treatment (Sun et al., 2008). The results of this paper suggested that bryostatin-1 was able to prevent ischemia-induced synaptic loss through promoting synaptogenesis.

Paralysis is a major sequel of stroke, which is difficult to recover. Monoaminergic or monoamine-related drugs such as amphetamine, levodopa, and serotonin reuptake inhibitors have been shown to be beneficial in stroke rehabilitation through increasing monoamine levels in the brain (Scheidtmann et al., 2001; Chollet et al., 2011). In the rats with cerebral cortex infarctions induced by photothrombosis, exercise improved the performance of rats in rotarod test, and co-administration of bryostatin-1 further enhanced the improving effect of exercise on functional motor recovery (Mizutani et al., 2016). Eight days after infarction, the serotonin concentration in perilesional cortex of rats treated with exercise and bryostatin-1 was markedly higher than that of rats treated with exercise alone. Conversely, serotonin turnover was markedly lower in the combination group. In addition, walking latency in the rotarod test showed a significant positive correlation with serotonin level, indicating that bryostatin-1 may improve motor function through regulating monoamine levels (Mizutani et al., 2016). Bryostatin-1 also increased 5-HT immunoreactivity in the dorsal raphe nucleus at 8 days after cerebral infarction (Mizutani et al., 2016). These results suggested that bryostatin-1 may be beneficial for paralysis recovery in stroke rehabilitation, which was possibly mediated by modulating monoamine levels in related brain regions.

### 2.5 Bryostatin-1 and traumatic brain injury

Traumatic brain injury (TBI) is a main cause of brain dysfunction characterized by structural damage and cell loss in the brain, which may result in permanent disability or death. The pathologies underlying TBI are poorly understood and the treatment modalities are limited.

PKC isozymes have been reported to translocate to the plasma membrane within 3 h after TBI and remain active for days (Yang et al., 1993; Muscella et al., 2008). The increase in PKCα activity contributes to mitochondrial dysfunction and secondary neuronal injury through glutamate receptor-mediated calcium oscillations (Yang et al., 1993; Geddes-Klein et al., 2006). However, PKCε can reduce vessel tone and produce neuroprotective effects. BBB disruption is an important indicator of brain injury and contributes to long-term and diffuse neuroinflammation (Perez-Polo et al., 2013). In a blast-induced TBI rat model, bryostatin-1 significantly attenuated the BBB breakdown caused by blast exposure. The mechanism study revealed that bryostatin-1 increased the levels of tight junction proteins (e.g., cadherin, ZO-1, and occludin) through down-regulating the toxic PKCα level and up-regulating the neuroprotective isozyme PKCε (Lucke-Wold et al., 2015).

In the genetically engineered human *Apolipoprotein E4* (*APOE4*) targeted replacement (TR) mice, two doses of 20 μg/kg bryostatin-1 treatment (i.p.) significantly reduced the TNF-α level and inhibited microglia activation in the cortex induced by repeated mild TBI (rmTBI) (Giarratana et al., 2020). Bryostatin-1 administration for 1 week improved the fine motor balance skills of APOE4 mice detected by balance beam test, but did not affect the gross vestibular motor function (Giarratana et al., 2020). Five injections of bryostatin-1 over a period of 14 days attenuated the learning and memory deficits induced by TBI as well as increased the expression of pre-synaptic synaptophysin and post-synaptic spinophilin (Zohar et al., 2011). The mechanism study found that bryostatin-1 protected against mTBI-induced cognitive and synaptic dysfunction possibly by increasing the activity of putative α-secretase ADAM10 and reducing β-secretase activity (Zohar et al., 2011). The above researches laid a foundation for further studying the effects of bryostatin-1 on TBI.

### 2.6 Bryostatin-1 and depression

Major depressive disorder (MDD) is one of the most prevalent neuropsychiatric disorders in humans, with a high risk of disability and mortality. Selective serotonin reuptake inhibitors (SSRIs) are the first-line drugs for treating depression. However, they are only effective for a part of patients and have obvious side effects. Other considerable number of patients with MDD shows limited reaction to the available antidepressants (Ionescu et al., 2015). Therefore, it is still urgent to explore new potential antidepressants.

Intravenous injection of bryostatin-1 through the tail vein at 20 μg/m^2^ (two doses every week for 5.5 weeks) significantly inhibited the immobility of depressive rats and also attenuated the deficits of spatial learning and memory in the rats after depressive behavior induction (Alkon et al., 2017). Another study also showed that bryostatin-1 at higher (100 nmol/kg, i.v.) and lower dose (32 nmol/kg, i.v.) both produced evident antidepressive effects by reducing non-searching immobility in depressive rats, which can be largely blocked by co-administration of a PKC inhibitor. It suggested that PKC activation is involved in the antidepressant function of bryostatin-1 (Sun and Alkon, 2005). The “vascular depression” refers to the depression caused by brain ischemic lesions resulted from cerebrovascular diseases. The acute administration of bryostatin-1 (20 μg/m^2^, two doses) through the tail vein at the induction phase of “ischemic depression” was shown to prevent the occurrence of depression caused by cerebral ischemia (Sun and Alkon, 2013). After inducing depressive behavior, chronic administration of bryostatin-1 (i.v., 20 μg/m^2^, twice a week for 5 weeks) through the tail vein also reversed the depressive immobility and this antidepressant effects could last for at least 3 weeks after discontinuation of the treatment (Sun and Alkon, 2013). These data suggested that bryostatin-1 and/or its analogs have the great potential to be developed as new antidepressants.

### 2.7 Summary of the neuropharmacological activities of Bryostatin-1

**FIGURE 2:**
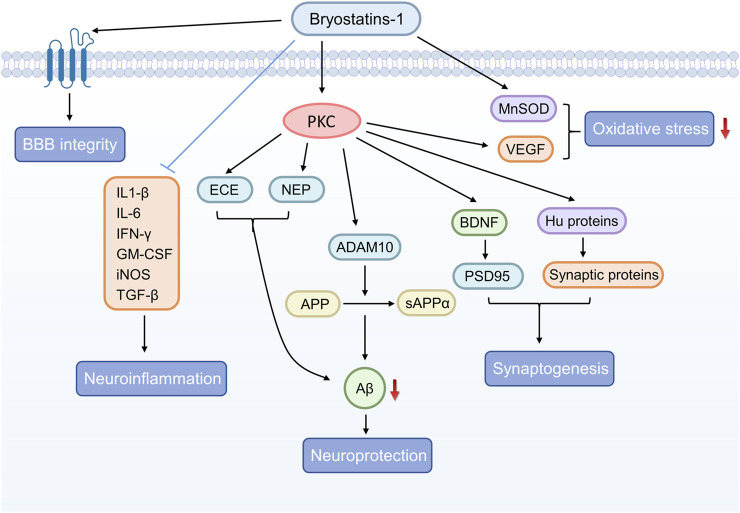
The graphical summary of signaling pathways underlying the neuroprotective effects of bryostatin-1.

The pharmacological activities of bryostatin-1 in the related neurological disorders are shown in Table 1 and the potential signaling pathways underlying the neuroprotective effects of bryostatin-1 are summarized in Figure 2.

**TABLE 1:** Pharmacological activities of bryostatin-1 in neurological disorders.

Disease	Study subject	Dose	Results	References
Alzheimer’s Disease	Tg2576 mice	Administration of 30 μg/kg (i.p.) twice a week for 12 weeks	↑Levels of PKCα, ε, and BDNF	Hongpaisan et al. (2011)
			↓The level of soluble Aβ	
			↓Synaptic loss	
	APP/PS1 mice	5 μg orally for 3 days during the first week and daily during the second week	↓Latency and distance to find the escape platform in Morris water maze test	Schrott et al. (2015)
	Tg2576 mice	30 μg/kg (i.p.) for 12 weeks	↑Levels of PKCɛ and MnSOD	Sen et al. (2018)
			↓oxidative stress	
	Aged rats and Tg2576 mice	30 μg/kg (i.p.) twice a week for 14 weeks	↑Levels of PKCε, MnSOD and VEGF	Millien et al. (2022)
			• Bryostatinprevented microvascular loss and age-related memory impairment	
	Brown Norway rats	5 μg/kg (i.p.), 30 min after water maze training on days 1, 3, and 5	↑The amplitude and frequency of sIPSCs	Xu et al. (2014)
			↑The firing rate of GABAergic interneurons	
			↑The paired-pulse ratio of GABAergic synapses	
	Advanced AD patients	i.v. (45 ± 5 min, with a total of 7 doses) over the course of 12 weeks	the SIB comparison favored 20 μg bryostatin compared to placebo patients in the Completer Analysis Set (CAS)	Farlow et al. (2019)
	AD patients	Single injection of bryostatin-1 (i.v.) at 25 μg/m^2^	↑The MMSE score at 3 h of injection	Nelson et al. (2017)
	Advanced AD patients	i.v. (45 ± 5 min, with a total of 7 doses) over the course of 12 weeks	improvement of 4.0 points or more in the SIB	Thompson et al. (2022)
Multiple sclerosis	Experimental autoimmune encephalomyelitis C57BL/6 mice	15 μg/kg (i.v.) for 15 days from the peak of disease	↓The percentage of demyelination area	Wu et al. (2022a)
			↓The total number of mononuclear cells	
			↓The proportions and absolute numbers of CD4^+^ cells	
			↓Astrogliosis in the spinal cord	
			↓M1 phenotype, ↑M2 phenotype	
			•Bryostatin-1 suppress CNS inflammation	
	C57BL/6 mouse model induced by cuprizone	15 μg/kg (i.v.) for 2 weeks	↑The area of myelination	Wu et al. (2022b)
			↑Protection ability of the myelin sheath	
			↑The myelin thickness of remyelinated axons	
			↓The activation of astrocytes	
			↓Levels of iNOS, claudin-5, ↑Arg1	
FXS	Fmr1 KO2 mice	20 μg/m^2^ (i.v.), two doses every week for 13 weeks	↓Hyperactivity	Cogram et al. (2020)
			↑Habituation to a novel environment	
			↓Impairments of memory	
	fragile X mice	20 μg/m^2^ (i.v.), 2 doses/week for 13 weeks	↑BDNF Levels, PSD-95, andGSK-3b Phosphorylation	Sun et al. (2014)
			↑Spatial Learning and Memory	
Stroke	MCAO aged female rats	2.5 mg/kg (i.p.) at 2 h and r-tPA at 6 h after the surgery	↓Mortality, hemispheric swelling	Tan et al. (2015)
			↓MMP-9 activation; ↑PKCε	
	MCAO rats	2.5 mg/kg (i.p.) every 3 days for a total of 7 doses over 21 days	↑Survival rates	Tan et al. (2013)
			↓Latency speed and distance traveled in Morris water maze test	
			↓Lesion volume of cortex, striatum, and total hemisphere	
	Global cerebral ischemic rat	15 μg/m^2^ (i.v.), two doses/week for 5 weeks	↓Pathophysiological molecular cascades and apoptosis	Sun et al. (2009)
			↑Neurotrophic activity; ↑BDNF activity	
	cerebral cortex infarcted rats	10 μg/m^2^ (i.v.), 5 days after infarction	↑Serotonin concentration in perilesional cortex	Mizutani et al. (2016)
			↑5-HT immunoreactivity in the dorsal raphe nucleus	
Traumatic brain injury	TBI model rats	2.5 mg/kg (i.p.), 5 min after blast exposure	↑PKCε; ↓BBB breakdown	Lucke-Wold et al. (2015)
			↑Levels of VE-cadherin, ZO-1, and occluding	
	APOE4 TR mice	Two doses of 20 μg/kg (i.p.), 5 min/24 h after the final injury	↑TNF-α level	Giarratana et al. (2020)
			↓Microglia activation in the cortex	
	TBI model rats	30 μg/kg (i.p.), 5 dose for 14 days	↓Learning and memory deficits	Zohar et al. (2011)
			↑Expression of pre-synaptic synaptophysin and post-synaptic Spinophylin	
			↑Levels of ADAM10	
Major depressive disorder	Depressive rats induced by open space swim test	20 μg/m^2^ (i.v.), 2 doses/week for 5.5 weeks	↓The immobility time in the forced swimming test	Alkon et al. (2017)
			↓Learning and memory deficits	
	Depressive rats induced by open space swim test	100 nmol/kg; 32 nmol/kg (i.v.), 3 h before the second trial	↓Non-searching immobility	Sun and Alkon (2005)
			↑Distance moved in open space swim test	
	Global cerebral ischemia mice induced by 2-VO method	20 μg/m^2^ (i.v.), twice a week for 5 weeks	↓Depressive immobility	Sun and Alkon (2013)

## 3 The pharmacokinetics of bryostatin-1

Although the data of human pharmacokinetics is rare because of lacking highly sensitive bio-analytical methods, the acceptable mice pharmacokinetics has been reported (Raghuvanshi and Bharate, 2020). The pharmacokinetic characteristics of bryostatin-1 administrated by i.p. and i.v. in mice have been compared with radiolabelled method (Zhang et al., 1996). Following administration of bryostatin-1 (i.p.) at a dose of 40 μg/kg, its plasma disappearance curve was approximately in line with a first-order absorption one-compartment model, and the absorption half-life and elimination half-life was 0.81 h and 28.76 h, respectively. However, the plasma disappearance curve for bryostatin-1 was in line with a two-compartment model after being administrated by i.v., with half-lives of 1.05 and 22.96 h (Zhang et al., 1996). The short half-life suggested that bryostatin-1 was rapidly distributed from the plasma to other tissues with both i.p. and i.v. administration, and the elimination half-life (nearly 29 and 23 h) illustrated that bryostatin-1 could stay in the body for a long time. The maximum plasma concentration (C_max_) of bryostatin-1 was 13.5 ng/mL after being given by i.p., which was lower than that of i.v. administration (92 ng/mL). However, the AUC of treatment by i.p. (620 ng/ml.h) was greater than that of i.v. (376 ng/ml.h) (Zhang et al., 1996). In the first 0.5 h after i.v., bryostatin-1 was distributed quickly in a variety of tissues with the highest concentration being found in the liver, lung, and bone marrow. The concentrations of bryostatin-1 in these tissues were significantly higher than that of plasma 4 h after administration. Bryostatin-1 was stable and largely remained intact in these organs. Following administration by i.p., bryostatin-1 was distributed in a similar pattern with that of i.v. From 6 h after administration, the concentrations of bryostatin-1 in most tissues were comparable or higher than those observed with i.v. injection at similar time points. During the first 12 h of post-administration, bryostatin-1 was eliminated from the body mainly through urinary excretion. However, significant fecal excretion was also observed within 72 h after dosing (Zhang et al., 1996). It indicated that urinary and fecal excretion were the major pathways for eliminating bryostatin-1. These data suggested that bryostatin-1 was well absorbed, distributed into and retained in tissues, and relatively slowly excreted after administration.

Nelson *et al* investigated the pharmacokinetic characteristics of bryostatin-1 in the brain. Administration of bryostatin-1 via tail vein at the dose of 10 and 15 μg/m^2^ (equivalent to 3.50 and 5.25 μg/kg) yielded similar brain concentrations with a maximum concentration of 0.20 nM in mice (Nelson et al., 2014). It indicated that the brain uptake of bryostatin-1 was saturated at doses of 10 μg/m^2^. The brain concentrations of bryostatin-1were 42% and 30% of the respective plasma concentrations 4 h after administration at the two doses, and the peak brain concentrations were 15.3% and 8.1% of the peak plasma concentrations respectively. The half-life of bryostatin-1 in brain was estimated to be above 10 h (Nelson et al., 2014). These data suggested that a considerable part of bryostatin-1 could cross the blood-brain barrier and enter the brain after peripheral administration despite its large molecular weight. Brain PKCε activation was biphasic which reached the peak at 0.5 h after bryostatin-1 administration and then slowly dropped to resting levels, even as the brain concentration of bryostatin-1 continued to increase. The effect of bryostatin-1 on brain PKC translocation was also biphasic, with maximal effects observed at 30–120 min and doses ranging from 5 μg/m^2^ to 10 μg/m^2^. In addition, only PKCε translocation was observed in the mouse brain, indicating the higher affinity with ε than other subtypes of PKC (Nelson et al., 2014). A similar result was also observed by *Zohar et al*, bryostatin-1 could cross the BBB and its brain concentration was nearly about half of the blood concentration and this ratio remained relatively stable within the first 24 h of injection (Zohar et al., 2011).

## 4 Safety and toxicity

The safety profile of bryostatin-1 has been investigated in some preclinical and clinical studies. Zhu *et al* examined the developmental toxicity of bryostatin-1 in pregnant rats during the sensitive teratogenesis period (Jiangbo et al., 2010). The rats were administered with 4.0, 8.0, and 16.0 μg/kg bryostatin-1 through tail vein during gestational day 6–15 once a day. When pregnant rats were treated with 8.0 and 16.0 μg/kg bryostatin-1, the weight of the conceived rats was markedly lower than control animals, suggesting that bryostatin-1 had maternal toxicity in rats when given above the dose of 8.0 μg/kg. The mortality of embryos, i.e., resorption of embryos and death of fetus rates were remarkably higher in animals treated with 16.0 μg/kg bryostatin-1, indicating that bryostatin-1 was embryotoxic at the dose above 16.0 μg/kg (Jiangbo et al., 2010). The fetal weights and body lengths in all three groups treated with bryostatin-1 were significantly lower than that of control animals, illustrating that bryostatin-1 was fetotoxic at the dose above 4.0 μg/kg (Jiangbo et al., 2010). These data shows that exposure to bryostatin-1 is toxic during organogenesis and it should be used with caution in pregnant women.

In a phase II study with advanced Alzheimer’s disease (AD) patients, bryostatin-1 (i.v.) at the dose of 20 μg for 12 weeks was shown to be safe and the patients in 20 μg treatment arm had a similar rate of adverse events (AE) compared to the placebo. However, there were more AEs such as diarrhea, headache, fatigue and myalgia among patients in the 40 μg (25 μg/cm^2^) arm (Farlow et al., 2019). In a phase II study with relapsed chemotherapy-resistant epithelial ovarian cancer, bryostatin-1 administered alone at a dose of 25 μg/m^2^ as a weekly 24-h infusion caused significant myalgia in nearly a half of patients (Clamp et al., 2003). In the patients with advanced renal cancer, the most common toxicities were myalgia and fatigue when treated with bryostatin-1 at a dose varying from 25 to 40 μg/m^2^, other common side effects included dyspnea, nausea, headache and vomiting (Haas et al., 2003; Madhusudan et al., 2003). Bryostatin-1 was also studied in combination with other anti-tumor drugs in different types of cancer at the dose ranging from 20 μg/m^2^ to 50 μg/m^2^ (Ku et al., 2008; Pavlick et al., 2009; Morgan et al., 2012; Plimack et al., 2014). Generally speaking, bryostatin-1 was well-tolerated and could be safely administered with those drugs with minimal toxicity (Pavlick et al., 2009). However, myalgia, the most common dose-limiting toxicity, may also preclude tolerability and prevent the combination (Ku et al., 2008; Morgan et al., 2012). The reported major toxicities in a previous paper summarizing the clinical trial results of bryostatin-1 were also myalgias, nausea, and vomiting (Kortmansky and Schwartz, 2003).

Several factors may affect the safety of bryostatin-1. First, the toxicity of bryostatin-1 may be closely related to the pathophysiological status of the drug users. The pharmacokinetic characteristics of bryostatin-1 are likely to be diverse in different disease states, which may lead to significant differences in absorption, distribution, metabolism and excretion of the drug in the body even at the same dosage. All these differences can influence the concentration and retaining time of bryostatin-1 in specific tissues, thereby affecting the safety of bryostatin-1. Second, the toxicity of bryostatin-1 is positively correlated with its dose. The higher dose of bryostatin-1 may predict a greater toxicity in theory. Third, drug combination is another important factor that cannot be ignored. There may be pharmacokinetic and pharmacodynamic interactions between bryostatin-1 and the combined drugs, which properly brings influence on the safety of bryostatin-1.

## 5 Conclusion and future perspectives

The sea is a great treasure of the earth and it conceives abundantly useful resources. Marine organisms are important sources of natural products and potential drug candidates for the clinic. In this review, the beneficial neuropharmacological effects of bryostatin-1, a bioactive marine macrolide from *B. neritina*, in both *in vitro* and *in vivo* studies were summarized. Bryostatin-1 showed promising effects in a variety of neurological disorders such as AD, MS, FXS, stroke, TBI and depression. This evidence suggests that bryostatin-1may be a suitable candidate for the treatment of neurological disorders. However, some limitations of this review have to be noticed. First, the preclinical experiments accounted for the majority of included studies, and the data on humans was rare. Bryostatin-1 was only successfully advanced to the clinical trial stage for treating AD among a series of neurological disorders. Whether it can be used to treat other CNS diseases is still unknown. It means that there is still a long way to go before the application of bryostatin-1 in treating neurological diseases. In addition, we mainly focused on the function of bryostatin-1in CNS in this paper, it is possible that bryostatin-1 has more potential for treating other diseases rather than neurological disorders. For example, bryostatin-1 displays encouraging profile in treating cancers in clinical trials when combining with other chemotherapy agents, such as cisplatin, paclitaxel, vincristine, gemcitabine. The combination of bryostatin-1 and paclitaxel has already been approved as an “orphan drug for esophagal cancer” (Raghuvanshi and Bharate, 2020).

Despite all this, the encouraging effect of bryostatin-1 in the clinical trials of AD makes us believe that it has a hope to be developed to treat relevant neurological disorders in the near future. But before taking bryostatin-1 a step closer to the clinic, there are still some important issues that needed to be addressed. First, the scalable supply of bryostatin-1 is a challenge because of its low natural abundance, as well as the environmental damage and economic cost associated with harvesting the marine organisms. Although *Wender et al* has successfully shorten the total synthesis of bryostatin-1 from initial 79 steps (Keck et al., 2011) to 29 steps now (Wender et al., 2017), its synthesis is still complex and the yield is low (with about 5% yield) (Wender et al., 2017). Second, despite the encouraging results in animal models of brain disorders, the molecular weight of bryostatin-1 is large and seems to lack the physicochemical features typically related with most successful CNS therapeutics (Wager et al., 2016). Although it can cross BBB, the peak concentration of bryostatin-1 in the brain is low (Nelson et al., 2014). Third, the human data of bryostatin-1 pharmacokinetics is rare because of the unavailability of highly sensitive bio-analytical methods (Raghuvanshi and Bharate, 2020). Fourth, higher dose of bryostatin-1 may cause the downregulation of PKC, so the therapeutic window of efficacy (the dose range) is an issue needed to be considered (Nelson et al., 2014). Fifth, although bryostatin-1 is generally well-tolerated, there are still drop-outs due to side effects (especially myalgia) in the clinical trials (Morgan et al., 2012). Therefore, overcoming the severe side effects of bryostatin-1 is another issue needed to be addressed in the future.

In view of the above-mentioned impediments, future research might be focused on the following directions: First, developing large-scale industrial synthetic routes of bryostatin-1 or synthesizing bioactive analogues close to bryostatin-1 in a step-economical manner. Second, develop highly sensitive bio-analytical methods to carry out human pharmacokinetic studies of bryostatin-1. The detailed human pharmacokinetic data will provide guidance for formulating more effective and safer drug regimens in clinic. Third, develop new drug delivery systems to increase the concentration of bryostatin-1 in the brain. Fourth, modify the relevant groups of the chemical structure of bryostatin-1 to reduce the potential side effects. Taken together, the promising neuropharmacological effects of bryostatin-1strongly indicate that it may be a potential candidate for treating neurological diseases, though some issues should be further addressed before expanding bryostatin-1 treatment into humans.
